# Congenital Neurological Disease Associated With HoBi-like Pestivirus Infection in a Newborn Dairy Calf From Brazil

**DOI:** 10.3389/fvets.2022.852965

**Published:** 2022-03-24

**Authors:** José Victor Pronievicz Barreto, Elis Lorenzetti, Juliana Torres Tomazi Fritzen, Andressa de Melo Jardim, Thalita Evani Silva Oliveira, Selwyn Arlington Headley, Amauri Alcindo Alfieri, Luiz Fernando Coelho da Cunha Filho

**Affiliations:** ^1^Post Graduate Program in Animal Health and Production, Department of Agrarian Sciences, Universidade Pitágoras Unopar, Arapongas, Brazil; ^2^Laboratory of Animal Virology, Department of Preventive Veterinary Medicine, Universidade Estadual de Londrina, Londrina, Brazil; ^3^Multi-User Animal Health Laboratory, Molecular Biology Unit, Department of Veterinary Preventive Medicine, Universidade Estadual de Londrina, Londrina, Brazil; ^4^Laboratory of Animal Pathology, Department of Veterinary Preventive Medicine, Universidade Estadual de Londrina, Londrina, Brazil; ^5^National Institute of Science and Technology, Dairy Production Chain (INCT-Leite), Universidade Estadual de Londrina, Londrina, Brazil

**Keywords:** dairy cattle, transplacental transmission, neurologic symptoms, *Pestivirus H*, HoBiPeV

## Abstract

HoBi-like pestivirus (HoBiPeV) has been reported in several biological samples from cattle worldwide, but there are no descriptions of this virus associated with neurological symptoms. This report described the first occurrence of neurological disease associated with HoBiPeV in a newborn dairy calf. A mixed-breed Holstein calf had severe neurological symptoms at birth and died at 21 days old. The tissue fragments (central nervous system (CNS), myocardium, liver, kidney, lung, intestine, and spleen) were submitted to reverse transcription (RT)–PCR assay for the partial 5'-untranslated region (5'UTR) and N-terminal autoprotease (N^pro^) gene of the pestivirus genome, and the CNS tissue fragments were submitted to histopathological and immunohistochemical evaluation. The RT–PCR assay indicated that the kidney, CNS, and intestinal tissue fragments were positive for the pestivirus 5'UTR, and the CNS and intestinal tissue fragments were positive for the pestivirus N^pro^ gene. Amplicons with high DNA quantification in the 5'UTR (CNS—cerebral cortex) and N^pro^ (CNS—cerebral cortex and intestine) RT–PCR assays were sequenced. The nucleotide (nt) sequence and phylogenetic analysis of the 5'UTR strain exhibited 93.6 to 99.4%, 85%, 89.4 to 89.9%, 85.1%, and 90.5 to 91.5% nt identity with HoBiPeV strains from clades a, b, c, d, and e, respectively. The N^pro^ amplicons showed 99.7% nt identity to each other and 90.4 to 96.5%, 85.1 to 85.3%, 79.2 to 79.7%, and 85.8 to 86.5% nt identity with HoBiPeV strains from clades a, c, d, and e, respectively. A histopathology revealed neuronal necrosis at the cerebrum, cerebellum, and brain stem. An immunohistochemical assay designed to identify antigens of bovine viral diarrhea virus revealed positive intracytoplasmic immunoreactivity within neurons at the cerebral cortex, cerebrum, cerebellum, and spinal cord. Thus, this report provides information about the first identification of HoBiPeV in tissues of the CNS in a newborn dairy calf with neurological symptoms.

## Introduction

The Pestiviruses comprise a genus within the *Flaviviridae* family and are currently classified into 11 species (*Pestivirus A to K*) (1). *Pestivirus A* (bovine viral diarrhea virus 1—BVDV-1), *Pestivirus B* (BVDV-2), and *Pestivirus H* (HoBi-like pestivirus—HoBiPeV) are the major pestivirus species infecting cattle (1). HoBiPeV was initially detected in commercial fetal bovine serum from Brazil by Schirrmeier et al. (2), and the viral strain was named D32/00 “HoBi.”

Pestiviruses are spherical enveloped viruses with a single-stranded positive-sense RNA. The genome of 12.5 kb consists of a long and single open reading frame encoding a 3,998 amino acid polyprotein (3) that can be cleaved into 11 to 12 polypeptides: N^pro^, C, E0/E^rns^, E1, E2, p7, NS23 (NS2-3), NS4A, NS4B, NS5A, and NS5B (4). The pestivirus 5'-untranslated region (5'UTR) contains 360 to 390 nucleotides (nt) (5) and is used for genetic characterization and phylogeny (6). The N-terminal autoprotease (N^pro^) coding region is variable in different species and subtypes of pestiviruses (7) and is used for genotyping wild-type virus strains (8, 9). Recently, based on the genomic 5'UTR and N^pro^ gene, the HoBiPeV strains have been classified into clades (a to e) (6, 9–11).

Infections caused by BVDV-1 and BVDV-2 have a worldwide distribution. Like HoBiPeV, they usually result in subclinical infections (12, 13) and may be associated with reproductive, respiratory, gastrointestinal, and/or hemorrhagic disorders (14). The antigenic and genomic differences of these viruses can interfere with diagnosis and vaccination (15, 16). The high mutation rate of HoBiPeV may explain the differences in its biology, virulence, and immunogenicity (17).

HoBi-like pestivirus (HoBiPeV) has been identified by reverse transcription-PCR (RT–PCR) assays performed on different types of biological material and in several countries, such as Bangladesh (18), Thailand (19, 20), India (9), and Italy (12). Occasionally, the genome of HoBiPeV was detected from the central nervous system (CNS) of a persistently infected (PI) calf with mucosal diseases-like in the absence of neurological symptoms (21); however, the association of HoBiPeV with neuropathological lesions was not confirmed by the immunohistochemical (IHC) assay.

Since the early 2000s, several studies have demonstrated the presence of HoBiPeV in biological samples from beef and dairy cattle herds from different geographic regions of Brazil, indicating that this virus may be endemic in this country (6, 13, 15, 22–26).

This case report describes the first occurrence of congenital neurological disease associated with HoBiPeV in a dairy calf.

## Materials and Methods

### Clinical History

A mixed-breed Holstein one-day-old calf with clinical manifestations of apathy, recumbency, and opisthotonos was examined at the Unopar Veterinary Teaching Hospital (Arapongas, Paraná State, Brazil). Clinical evaluation revealed motor incoordination, ataxia, abnormal menace response, and proprioceptive deficit, suggestive of cerebellar syndrome. However, there was no clinical improvement; the calf died suddenly after 20 days of hospitalization and was submitted for routine postmortem evaluation. There is no report of any previous disease at this herd; furthermore, cattle at this establishment were not vaccinated against infections by BVDV.

### Post-mortem Evaluations

A routine postmortem evaluation was performed after death. Tissue sections (CNS, myocardium, liver, kidney, lung, intestine, and spleen) were fixed by immersion in 10% buffered formalin solution and routinely processed for histopathological evaluation with the hematoxylin and eosin staining. Duplicate tissue fragments were collected for the IHC assay, while freshly collected samples were maintained at −80°C until use in molecular assays.

### Molecular Investigations

Tissue fragments (cerebral cortex, cerebellum, spinal cord, rete mirabilis, myocardium, liver, kidney, lung, intestine, and spleen) were processed with 0.01 M phosphate-buffered saline (137 mM NaCl, 3 mM KCl, 8 mM Na_2_HPO_4_, 14 mM KH_2_PO_4_; pH 7.2) in 10% suspension (w/v) and centrifuged at low speed for 5 min. The supernatant (500 μl) was recovered and pretreated at 56°C for 30 min with sodium dodecyl sulfate and proteinase K at final concentrations of 1% (v/v) and 0.2 mg/ml, respectively. The nucleic acid was extracted by combining the phenol/chloroform/isoamyl alcohol (25:24:1) and silica/guanidine isothiocyanate techniques (27). The nucleic acid was eluted in 50 μl of ultrapure diethylpyrocarbonate (DEPC)-treated water (Invitrogen Life Technologies, Carlsbad, CA, USA) and immediately stored at −80°C until use.

The extracted nucleic acid was submitted to RT–PCR assay using the panpestivirus primers (324, forward 5′ATGCCCWTAGTAGGACTAGCA3′ and 326, reverse 5′TCAACTCCATGTGCCATGTAC3′), designed to amplify a 288-base pair (bp) product from the 5′UTR of the pestivirus genome (28). Additionally, the primer pair designed to amplify a 428-bp length amplicon from the N^pro^ gene (BD1, forward 5′TCTCTGCTGTACATGGCACATG3′ and BD3, reverse 5′CCATCTATRCACACATAAATGTGGT3′) (8) was used to confirm the results of the phylogenetic analysis based on the 5′UTR fragment. The RT and PCR assays were performed according to Lunardi et al. (29) and Vilček et al. (8) for the 5′UTR and N^pro^ gene, respectively. Aliquots of ultrapure DEPC-treated water were included as a negative control, and the BVDV-1 cell culture-adapted NADL strain was included as the positive control. The RT–PCR products were analyzed by electrophoresis on 2% agarose gels in Tris-boric acid-ethylenediaminetetraacetic acid (EDTA) buffer, pH 8.4 (89 mM Tris; 89 mM boric acid; 2 mM EDTA), containing 0.5 μg/ml ethidium bromide. After electrophoresis at a constant voltage (100 V) for 40 min, the agarose gel was visualized under ultraviolet light.

Reverse transcription PCR (RT–PCR) amplicons were purified using the Illustra GFX PCR DNA and the Gel Band Purification Kit (GE Healthcare, Little Chalfont, Buckinghamshire, UK), quantified with a Qubit^®^ Fluorometer (Invitrogen Life Technologies, Eugene, OR, USA), and sequenced in an ABI3500 Genetic Analyzer sequencer using the same forward and reverse primers used in the RT–PCR assay with the BigDye^®^ Terminator v3.1 Cycle Sequencing Kit (Applied Biosystems, Foster City, CA, USA). The sequence quality analysis was carried out using the PHRED software, and contig assembly was obtained using the CAP3 software (http://asparagin.cenargen.embrapa.br/phph/). The nt sequences were compared with sequences deposited in the GenBank using the BLAST software (http://blast.ncbi.nlm.nih.gov/Blast.cgi). Phylogenetic trees based on the nt sequences were constructed using the neighbor-joining method and the Kimura two-parameter model in the MEGA software version 7.0.26. Bootstrapping was statistically supported with 1,000 replicates. The nt sequence identity matrices were performed in the BioEdit software version 7.2.6.1.

Four other etiologic agents that can also cause neurological clinical signs in calves were investigated by PCR assays targeting the glycoprotein C gene of bovine alphaherpesvirus 1 (BoHV-1, 354 bp) and bovine alphaherpesvirus 5 (BoHV-5, 159 bp) (30); the tegument protein gene of ovine gammaherpesvirus 2 (OvHV-2, 422 bp) (31); and the pNC-5 gene of *Neospora caninum* (337 bp) (32).

### Immunohistochemical Identification of BVDV

Formalin-fixed paraffin-embedded (FFPE) tissue fragments of the cerebral cortex, cerebellum, and spinal cord of the calf were used in an IHC assay designed to identify the antigens of BVDV using anti-BVDV #15c-5 antibody, as previously described (33). Positive controls consisted of the FFPE tissue fragments known to contain antigens of BVDV from a previous study (33). Two negative controls were used: the first consisted of substituting the primary antibodies with their respective diluents; the second consisted of utilizing the primary antibodies on FFPE tissues with known negative immunoreactivity to BVDV derived from the study cited above. Positive and negative controls were included in each IHC assay.

## Results

### Pathological Findings

**Figure 1:**
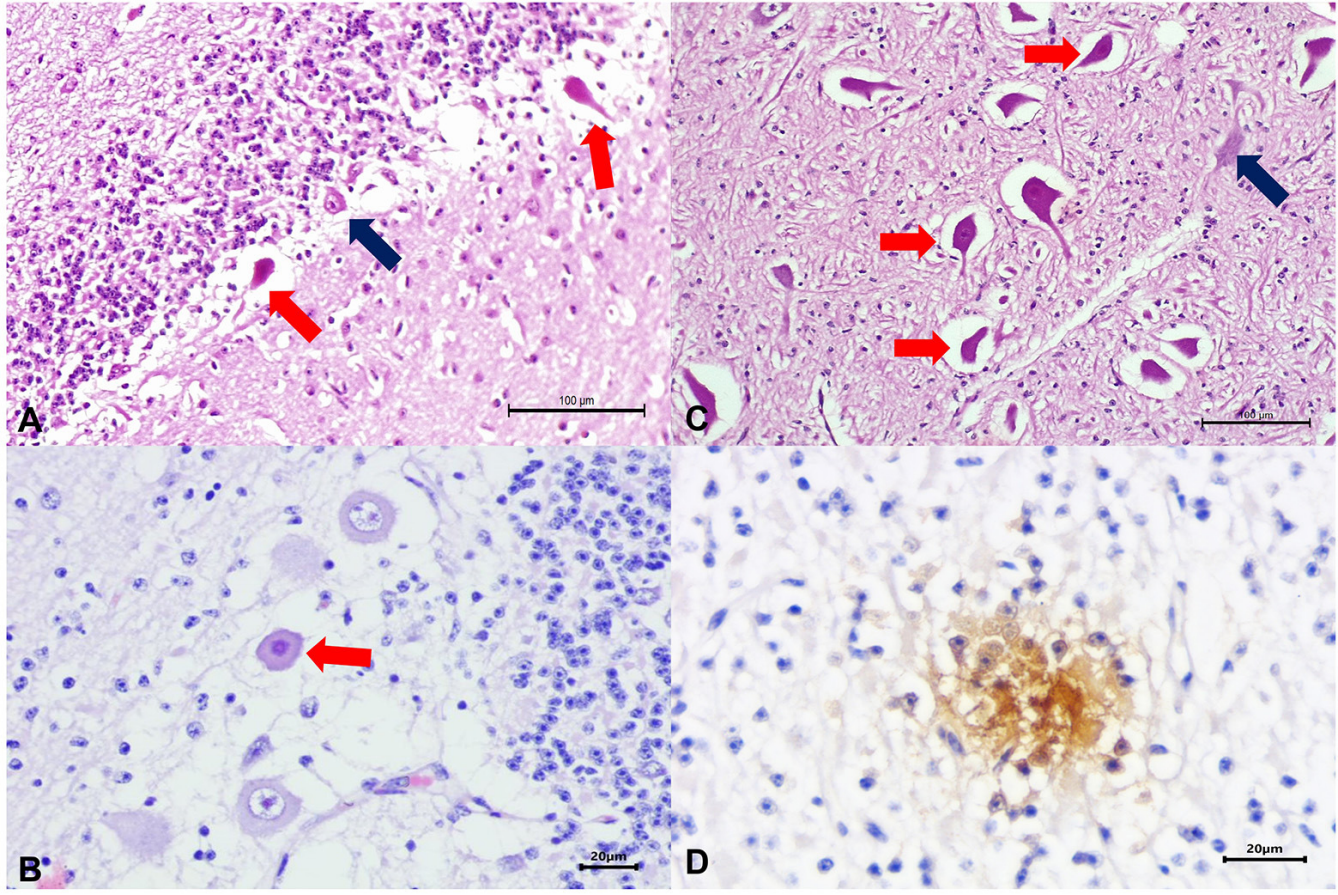
Histopathologic and immunohistochemical findings observed in a newborn calf naturally infected with HoBi-like pestivirus (HoBiPeV). There is necrosis of Purkinje cells at the cerebellum **(A,B)** and the brain stem **(C)**; compare the severely eosinophilic and shrunken necrotic neurons (red arrows) with the adjacent normal looking neurons (blue arrows). Observed positive intracytoplasmic immunoreactivity to antigens of bovine viral diarrhea virus (BVDV) within necrotic neuron of the cerebellum **(D)**. Hematoxylin and eosin stain **(A–C)**; immunoperoxidase counterstained with hematoxylin **(D)**. Scale bar A and C, 100 μm; B and D, 20 μm.

Significant gross lesions were not observed during routine postmortem evaluations. The histopathologic findings were predominantly neurological and seemed to affect neurons within several anatomic regions of the CNS, resulting in neuronal necrosis at the cerebrum, necrosis and degeneration of Purkinje cells of the cerebellum and brain stem (Figures 1A–C), and mild neuronal necrosis at the spinal cord.

### Molecular Findings

The RT–PCR assay amplified the 5′UTR of pestivirus from fragments of the cerebral cortex, cerebellum, spinal cord, rete mirabilis, kidney, and small intestine. Furthermore, the N^pro^ gene assay was positive in all tissues evaluated in the 5′UTR assay, except fragments from the kidney.

The nucleotide sequence identity analysis of the 5′UTR revealed that the amplicon derived from this study (GenBank accession n° MZ612417) had 93.6 to 99.4%, 85%, 89.4 to 89.9%, 85.1%, and 90.5 to 91.5% nt identity with HoBiPeV strains from clades a, b, c, d, and e, respectively, and 70.6 and 75.6% nt identity with BVDV-1 (NADL strain) and with BVDV-2 (890 strain), respectively.

Additionally, the nt sequence identity analysis of the N^pro^ amplicons (GenBank accession n° MZ612415 and MZ612416) obtained during this study had 99.7% nt identity to each other and 90.4 to 96.5%, 85.1 to 85.3%, 79.2 to 79.7%, and 85.8 to 86.5% nt identity with HoBiPeV strains from clades a, c, d, and e, respectively; and 67.3 to 67.5% and 66.3 to 66.5% nt identity with BVDV-1 (NADL strain) and with BVDV-2 (890 strain), respectively.

**Figure 2:**
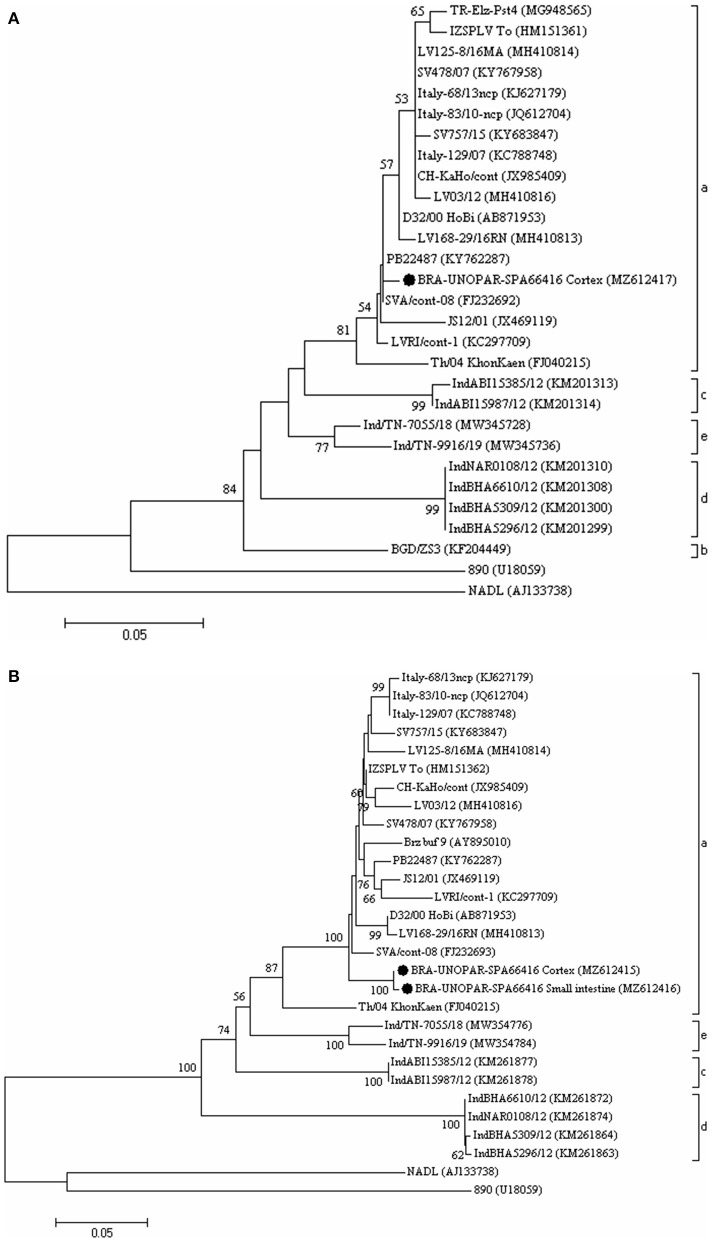
The phylogenetic analysis is based on the partial nucleotide (nt) sequences of the 5'-untranslated region (5'UTR) **(A)** and of the N^pro^ gene **(B)** of HoBiPeV strains described in this study. The trees were constructed using the neighbor-joining method and the Kimura two-parameter model for nt substitution. The bootstrap values are shown at the branch nodes (values <50% are not shown). The scale bars at the bottom of the trees represent the number of nt substitutions per site. The GenBank accession numbers of the strains are provided in parentheses. The HoBiPeV strains identified during this study are indicated with a filled circle. The strains used as outgroups were the BVDV-1 prototype strain NADL and BVDV-2 prototype strain 890.

The phylogenetic analyses of the 5′UTR (Figure 2A) and N^pro^ gene (Figure 2B) revealed that the wild-type strains identified in this study clustered with the HoBiPeV clade a strains.

Furthermore, nucleic acids of BoHV-1, BoHV-5, OvHV-2, and *N. caninum* were not amplified from the tissue fragments evaluated.

### Immunohistochemical Findings

Immunohistochemistry (IHC) revealed positive intracytoplasmic immunoreactivity for BVDV antigens within degenerated and necrotic neurons of the cerebral cortex, cerebellum, and spinal cord (Figure 1D).

## Discussion

The clinical signs presented by the calf described in this study were strictly neurological, characterizing a congenital cerebellar syndrome, different from those observed in cattle with HoBiPeV infection (9, 11, 12, 21). There are reports of CNS infection by BVDV-1 (34) and BVDV-2 (35, 36) by transplacental transmission with neurologic symptoms in cattle. Passler et al. (37) performed an experiment in which pregnant goats were infected with BVDV-1 and BVDV-2 strains to assess transplacental transmission and the degree of damage to the fetuses. BVDV-2 caused greater damage, and IHC analyses demonstrated the presence of the virus in fetal CNS tissue. The results herein described are similar to those reported by Passler et al. (37), suggesting that HoBiPeV can also have vertical transmission, causing damage to the fetuses, since the one-day-old calf had severe neurological symptoms associated with CNS tissue infection.

HoBi-like pestivirus (HoBiPeV) was first identified in Italy in 2004 from a batch of bovine fetal serum from Brazil (strain D32/00_Hobi) (2). HoBiPeV has been identified in fragments of different organs of cattle with and without clinical signs (12, 21, 22, 24, 25). This study represents the first detection of HoBiPeV causing neurological symptoms. Although Decaro et al. (21) reported the HoBiPeV genome coding the NS2-3 polypeptide by real-time RT-PCR (qPCR) assay in the brain, cerebellum, and brain stem of a PI calf that displayed severe mucosal disease without neurological symptoms, IHC was not performed, so the association of the neuropathological lesions with the viral infection was not confirmed.

Our study detected HoBiPeV RNA in fragment tissues by RT–PCR assay for the 5′UTR and N^pro^ gene. Cruz et al. (25) detected HoBiPeV in the tissue fragments of the spleen, lymph nodes, lung, liver, serum, and kidney using the RT–PCR assay for the 5′UTR and N^pro^ gene from two necropsied calves from an outbreak of the mucosal disease in a Brazilian beef cattle herd. However, they did not report neurological symptoms. Similarly, other studies conducted in Brazil have also reported the presence of HoBiPeV in serum samples and fragments of organs other than the CNS of cattle (6, 13, 15, 22, 23, 25, 26, 38).

This report analyzed the 5′UTR (GenBank accession no. MZ612417) and N^pro^ (GenBank accession no. MZ612415 and MZ612416) sequences from the HoBiPeV strains that were detected in a calf with neurological symptoms belonging to clade a. This clade was previously reported in bovine biological samples, not in CNS, based on previous studies performed in Brazil (6, 10). The HoBiPeV strains from clade b originated from Bangladesh (18), while the other three clades (c, d, and e) were described in India (9, 11). Despite the high frequency of diagnosis of HoBiPeV in Brazil, to date, only the HoBiPeV strains that belong to clade a were identified in this country.

Pestiviruses commonly cause cerebellar hypoplasia, and animals are born showing cerebellar syndrome with dysfunction to regulate and coordinate the motor activity and degeneration of Purkinje cells of the cerebellum as the main histological alteration (39). The calf from this report had no gross evidence of cerebellar hypoplasia, but presented a cerebellar syndrome due to degeneration of Purkinje cells, configuring an atypical manifestation of cerebellar syndrome caused by pestivirus. Histological findings described by Decaro et al. (21) in the brain of a PI calf with HoBiPeV that displayed severe mucosal disease were restricted to neurophagy and spongiosis of the gray cortex. These lesions were less severe than those described in this report, where the calf died shortly after showing severe and irreversible neurological symptoms.

The IHC performed by Cruz et al. (25) using the D89 monoclonal antibody showed positive immunoreactivity for BVDV for the distal limbs, gingiva, tongue, and esophagus of two PI calves by HoBiPeV. On the other hand, this report demonstrated positive immunoreactivity for antigens of BVDV in the cerebral cortex, cerebellum, and spinal cord, confirming the association of neuropathological lesions with a viral infection. Additionally, Marques et al. (24) confirmed the presence of HoBiPeV in the analysis of skin biopsies and ear notches by IHC using the same anti-BVDV #15c-5 antibody applied in this study.

The association of clinical signs, histopathologic and IHC findings, molecular detection and characterization confirmed the participation of HoBiPeV as the etiological agent associated with neurological symptoms observed in this newborn dairy calf. This report describes congenital HoBiPeV infection and contributes to the understanding of the etiopathogenesis of this infection in cattle. To the best of our knowledge, this is the first report of this pestivirus species causing neurological symptoms in a newborn calf.

## Data Availability Statement

The sequences determined in this study can be found in the GenBank database under the accession numbers MZ612415 to MZ612417. https://www.ncbi.nlm.nih.gov/genbank/.

## Ethics Statement

The animal study was reviewed and approved by Institutional Ethics Committee of Universidade Pitágoras Unopar (Protocol Code 023/17, June 22, 2017). All applicable international, national, and/or institutional guidelines for the care and use of animals were followed.

## Author Contributions

JB, EL, AA, and LC: conceptualization, validation, formal analysis, investigation, visualization, supervision, project administration, and data curation. JB, EL, JF, TO, SH, AA, and LC: methodology. EL: software. AA and LC: resources. JB, EL, JF, SH, AJ, and LC: writing—original draft preparation. JB, EL, SH, AA, and LC: writing—review and editing. AA and LC: funding acquisition. All authors have read, critically analyzed, approved the final draft of this manuscript, and have agreed to be accountable for all aspects of the study in ensuring that questions related to the accuracy or integrity of any part of the work are appropriately investigated and resolved.

## Funding

This research was funded by the following Brazilian institutes: National Foundation for the Development of Private Higher Education (FUNADESP), Brazilian Federal Agency for Support and Evaluation of Graduate Education (CAPES), National Council of Scientific and Technological Development (CNPq), Araucaria Foundation (FAP/PR), and National Institute of Science and Technology of Dairy Production Chain (INCT-Leite) [Grant Number 465725/2014-7].

## Conflict of Interest

The authors declare that the research was conducted in the absence of any commercial or financial relationships that could be construed as a potential conflict of interest.

## Publisher's Note

All claims expressed in this article are solely those of the authors and do not necessarily represent those of their affiliated organizations, or those of the publisher, the editors and the reviewers. Any product that may be evaluated in this article, or claim that may be made by its manufacturer, is not guaranteed or endorsed by the publisher.
